# BACK TO BASICS: HOPELESSNESS AS THE MEDIATING FACTOR FOR COMPLETED SUICIDE AMONG OLDER ADULTS

**DOI:** 10.1093/geroni/igz038.1001

**Published:** 2019-11-08

**Authors:** Silvia C Hernandez, James C Overholser, James Lavacot, Kristie L Philips, Craig A Stockmeier

**Affiliations:** 1 Case Western Reserve University, Cleveland, Ohio, United States; 2 Cleveland VA Medical Center, Cleveland, Ohio, United States; 3 University of Mississippi Medical Center, Jackson, Mississippi, United States

## Abstract

Individuals 65 years and older are at high risk for completing suicide. Though risk factors have been established in the literature, the dominant atheoretical approach has left the field at an impasse. The present study aimed to integrate core risk factors of hopelessness, depression, physical illness, and social isolation by proposing a biopsychosocial framework of older adult suicide. A psychological autopsy was used to compare individuals 65 years and older who died either by suicide (n = 32) or natural causes (n = 45). Structural equation modeling results suggested that hopelessness was the only factor directly associated with suicide (B = .01, β = 0.84, SE = 13.31, p ≤ .001), fully mediating the relationships between suicide and social isolation, negative attitudes about physical health, and depression. The proposed model adequately fit the data, explaining 71% of the variance in cause of death. Advanced age (75+ years) moderately increased social isolation, which weakly increased hopelessness, contributing to suicide in a smaller magnitude than expected. Though individuals in the advanced age group had a wider range of physical illnesses, this did not increase risk. Rather, negative perceptions of health increased risk for all individuals 65 years and older via depression and hopelessness, irrespective of the presence of impairing physical illness. Findings support the claim that hopelessness plays a pivotal role in the progression from suicidal ideation to completion among older adults. Directly targeting hopelessness could help prevent at-risk older adults from acting on their thoughts of suicide.

